# Identification and characterization of a novel inhibitor of influenza A virus that acts by blocking nucleoprotein oligomerization

**DOI:** 10.1128/aac.01149-25

**Published:** 2025-12-19

**Authors:** Vincent H. J. Leonard, Dianna B. Vidales, Benjamin R. Taft, Matthew J. Hesse, Patrick S. Lee, Mulugeta Mamo, Dirksen E. Bussiere, Karen C. Wolff, Kelli L. Kuhen, Laura Wedel, Ellena Growcott, Colin Osborne, Cassio P. Octaviani, Pinghan Huang, Chien-Te Kent Tseng, Johanna R. Abend, Kelly A. Wong, Weidong Zhong, David C. Tully, Don Ganem

**Affiliations:** 1 Novartis Institutes for BioMedical Research, Infectious Diseases 98558 , Emeryville, California, USA; 2 Novartis Institutes for BioMedical Research, Global Discovery Chemistry 98558 , Emeryville, California, USA; 3 Via Nova Therapeutics 706214 , Oakland, California, USA; 4 Genomics Institute of the Novartis Research Foundation 70089, San Diego, California, USA; 5 Department of Microbiology and Immunology, University of Texas Medical Branch 12338, Galveston, Texas, USA; Chinese Academy of Medical Sciences & Peking Union Medical College, Beijing, China

**Keywords:** antiviral, nucleoprotein, influenza

## Abstract

Influenza A virus (IAV) causes annual epidemics and sporadic pandemics of acute respiratory infections resulting in significant morbidity and mortality. Although approved influenza antivirals (e.g., oseltamivir and baloxavir) exist, concerns persist about the potential for emergence of drug-resistant variants, highlighting the continuing need for new antiviral therapies. Here, we describe the development of an orally bioavailable, direct-acting antiviral (VNT-101) with a novel mechanism of action: disrupting homo-oligomerization of the influenza nucleoprotein (NP) and thereby inhibiting viral RNA synthesis. Selection of drug-resistant mutants revealed amino acid substitutions mapping to the oligomerization domain of NP, and X-ray crystallography co-structure determination of VNT-101 complexed with recombinant NP confirmed VNT-101 binding in the oligomerization pocket. Biochemical experiments using size exclusion chromatography confirmed disruption of oligomerization when this chemotype is added to preparations of recombinant NP *in vitro*. VNT-101 has potent and specific activity against the currently circulating IAV subtypes H1N1 and H3N2, with mean EC_50_ values ranging from 2 to 18 nM, and displays strong efficacy in a murine model of lethal influenza infection when administered either prophylactically or therapeutically. Importantly, VNT-101 remains active against influenza variants that are resistant to either oseltamivir or baloxavir and also has potent activity against highly pathogenic avian H5N1 and H7N9 isolates that have transmitted to humans and represent strains of potential pandemic concern. These studies support the continued development of VNT-101 to augment our therapeutic arsenal against both seasonal and pandemic influenza.

## INTRODUCTION

Influenza is a seasonal respiratory viral infection that typically affects 8–10% of the population every year and causes significant morbidity and mortality. The Centers for Disease Control and Prevention estimates that influenza results in 100,000–710,000 hospitalizations and 4,900–51,000 deaths annually in the USA alone (1, 2). Additionally, global influenza pandemics occur every few decades when radically distinct influenza virus strains emerge for which people have no effective pre-existing immunity, resulting in >25% of the population becoming infected and increased levels of morbidity and mortality. Influenza is caused by two related types of orthomyxovirus: influenza A virus (IAV) and influenza B virus (IBV). IAV is the dominant pathogen in the family, accounting for ~85% of seasonal influenza cases and essentially all influenza pandemics (3).

IAV is a segmented, negative-strand RNA virus whose replication depends upon a multi-subunit RNA-dependent RNA polymerase. The viral RNA is complexed with the influenza nucleoprotein (NP), which is required for the presentation of the RNA template to the viral polymerase via interaction with polymerase subunits PB1 and PB2. NP exists as a multimer, and its multimeric nature is essential for its function. In the absence of functional NP, no viral RNA synthesis occurs (4, 5). NP also plays key roles in the transport of viral ribonucleoprotein (RNP) into and out of the infected cell nucleus (4).

Vaccination is the primary method of prevention and control of infection. Although vaccines for IAV are widely recommended, they are of only limited efficacy (approximately 40–65%), especially in the elderly and the immunocompromised (3), indicating the need for additional forms of intervention. Two classes of drugs have been approved for the treatment of IAV infection: influenza neuraminidase (NA) inhibitors (NAIs) (e.g., oseltamivir and zanamivir) and endonuclease inhibitors (of which there is only a single approved example, baloxavir). The clinical benefit of antiviral treatment is greatest when initiated soon after the onset of infection. These drugs shorten the duration of clinical symptoms by about 24 h in adult patients, but only if taken within 48 h of the onset of symptoms (3, 6, 7).

As for many antivirals, mutations in the viral targets of NAIs and endonuclease inhibitors can confer varying degrees of resistance. Since oseltamivir is the mainstay of influenza treatment, the emergence of resistance to this drug (and other NAIs) is a matter of some concern (3, 8). Oseltamivir resistance was rare in the first decade of its use. However, in 2007–2008, high-level resistance began to appear among H1N1 strains of IAV and spread rapidly. By early 2009, most circulating H1N1 strains in the USA and Europe were resistant (9). Then, in an unanticipated development, a global pandemic in the latter half of 2009 caused by a novel, oseltamivir-sensitive H1N1 strain led to the displacement of the earlier, resistant H1N1 strain from its human niche, serendipitously extending the clinical utility of the drug. Although currently circulating IAV strains remain generally sensitive to oseltamivir, these events show how abrupt and unpredictable the occurrence of resistance can be, especially in light of the fact that oseltamivir resistance in 2007–2009 was not correlated with widespread use of the drug.

In 2018, baloxavir, an inhibitor of influenza polymerase acidic protein PA, an endonuclease, was approved in the USA and Japan for the treatment of uncomplicated influenza infections (10, 11). While more potent than oseltamivir as an antiviral, baloxavir has a low genetic barrier to development of resistance. In clinical trials, 1.5–3% of patients with H1N1 strains of IAV infection and 7–10% of those with H3N2 strains developed drug-resistant mutants in their respiratory secretions during post-treatment convalescence (with higher percentages observed in pediatric populations) (12–14). These and other observations (12) raise concerns that influenza strains resistant to baloxavir could persist and impair the future utility of this drug (3). Thus, there is an ongoing need for new anti-influenza drugs with novel mechanisms of action that will allow effective therapy if resistant strains become dominant. Additionally, combinations of current therapies with a new agent would be expected to suppress the emergence of resistance to both classes of antivirals and thus could extend their clinically useful lifespans.

In this report, we describe the characterization of VNT-101, an orally bioavailable, direct-acting antiviral inhibitor of IAV replication that was optimized from a hit identified by phenotypic screening. Genetic experiments identify its target as the influenza NP, and biochemical and structural studies indicate that it acts by inhibiting NP oligomerization. The distinct mechanism of action and the demonstrated activity of VNT-101 in animal models of IAV infection make it a promising candidate for further clinical development.

## RESULTS

### A phenotypic screen for inhibitors of IAV replication identifies a novel compound series

IAV encodes a neuraminidase, the activity of which is required for efficient spread of infection. The level of this enzymatic activity was used as a marker of the extent of IAV A/Puerto Rico/8/1934 (H1N1) replication in MDCK cells in a high-throughput screen of a library of 3.4 million compounds. Compounds that inhibited neuraminidase activity were identified as hits, and following triaging of the chemical matter, the highest priority hits were confirmed and optimized using an influenza cytopathic effect (CPE) assay to evaluate antiviral activity. A chemical series was identified that markedly increased cell viability (i.e., protected cells from virus-induced CPE) following infection of MDCK cells with IAV at a low multiplicity of infection (MOI) and incubation for 72 h. Early hit-to-lead compounds VNT-725 (median EC_50_ = 0.19 µM, CC_50_ >50 µM) and VNT-754 (median EC_50_ = 0.29 µM, CC_50_ >50 µM) were selected as representative compounds of this series based on their potent antiviral activity and high selectivity index (15). Extensive medicinal chemistry optimization efforts were then undertaken, involving the investigation of over 700 derivatives (15). From this population, we selected VNT-101 as the optimal candidate for further preclinical and clinical development.

### *In vitro* resistance selection studies with VNT-101 identify influenza nucleoprotein (NP) as the biological target

To elucidate the specific viral target and mechanism of action of VNT-101, *in vitro* resistance selection studies were conducted. Briefly, IAV isolate A/Puerto Rico/8/1934 (H1N1) was serially passaged in MDCK cells in the presence of increasing concentrations of VNT-101. Deep sequencing of the resulting viral variants was used to identify mutations in the target gene when compared to control sequences from virus passaged in the presence of DMSO. Mutations were found only in the influenza NP segment (Table 1). One of these mutations (A336T) increased in prevalence over time to a peak of >99% frequency at passages 5 and 6 (Table 1, top row). Additional mutations in NP were present at lower frequencies and did not persist over time. Mutations in other influenza genes were not detected at frequencies above those observed in the DMSO-treated control.

**TABLE 1:** Activity of VNT-101 against nucleoprotein mutations identified during *in vitro* resistance selection^*a*^

	WT	V194I	V270I	H334Y	A336T	L344S	G460R
Peak prevalence	n/a	17.2% (P3)	6.8% (P1)	6.3% (P1)	99.8% (P5)	18.4% (P1)	14.6% (P1)
EC_50_ (µM) in mini-genome assay containing indicated NP mutations							
VNT-101 (fold shift)	0.035 (n/a)	0.040 (1.1×)	0.037 (1.1×)	>50 (>1,428× )	>50 (>1,428× )	0.477 (13.6×)	1.691 (48.3×)
Nucleozin^*b*^ (fold shift)	0.349 (n/a)	0.423 (1.2×)	0.324 (0.9×)	0.564 (1.6×)	0.360 (1.0×)	0.380 (1.1×)	0.430 (1.2×)

^*a*^ Data from the mini-genome assay are expressed as the mean EC_50_ from three independent experiments. Fold shift is calculated as the ratio of EC_50_ with mutant NP / EC_50_ with WT NP. NP, nucleoprotein; WT, wild type; n/a, not applicable; P, passage number.

^*b*^ Nucleozin, RNP inhibitor acting through a distinct mechanism from VNT-101 (16).

To prove that these mutations confer drug resistance and to determine the level of reduced susceptibility to VNT-101, each identified mutant was engineered into an NP expression construct and individually tested in a mini-genome reporter assay (also known as the RNP assay). In this assay, wild-type or mutant NP genes are co-transfected with the cDNAs encoding the viral polymerase subunits and a minigenome bearing viral cis-acting sequences and luciferase reporter, in the presence or absence of graded concentrations of VNT-101. As shown in Table 1, four mutations (H334Y, A336T, L344S, and G460R) caused significant shifts in the EC_50_, indicating that these mutants confer reduced susceptibility to VNT-101. Mutations H334Y and A336T were notable for conferring high-level resistance (EC_50_ >50 µM). Two mutations, V194I and V270I, did not impact the EC_50_ of VNT-101 relative to the wild-type construct. These are conservative substitutions and may simply represent polymorphisms in NP. However, it should be noted that the minigenome assay reads out primarily on the RNA replicative functions of NP and does not read out on other functions of NP (e.g., nucleocytoplasmic transport of viral RNPs [4]; induction of mitophagy [17]). Therefore, it is conceivable that these mutations might instead affect those functions of NP.

Nucleozin, a tool compound that inhibits IAV by triggering the aggregation of NP and inhibiting its nuclear accumulation, was used as a control compound that targets the same viral protein (16). The nucleozin EC_50_ values were similar across all NP constructs tested. In particular, the highly VNT-101-resistant mutants H334Y and A336T remained fully sensitive to nucleozin, demonstrating that nucleozin and VNT-101 act through distinct mechanisms of action, despite targeting the same viral protein (Table 1). This conclusion is further supported by detailed biochemical and structural studies described below.

### Co-structure of VNT-101 complexed with influenza NP demonstrates binding in the oligomerization pocket

Multiple crystal structures of NP have been published from different influenza isolates and subtypes (18–20). The protein is composed of three domains: a head domain, a body domain, and a 28-amino acid flexible tail loop (residues 401–428). When recombinantly expressed in the absence of viral genomic RNA, NP forms oligomers, predominantly the trimeric species (18). Oligomerization is based on the insertion of the tail loop of one NP subunit into a pocket located in the body of an adjacent subunit, with which it makes multiple interactions (18). Two sequence elements have been identified as critical for NP oligomerization: residues 189–358 and residues 371–465 (21). Furthermore, the formation of a salt bridge between binding pocket residue E339 and tail loop residue R416 has been highlighted as essential for NP-NP interaction (22). Notably, the four residues identified by *in vitro* resistance selection study with VNT-101 (H334Y, A336T, L344S, and G460R) reside in the two sequence elements previously implicated in NP oligomerization (residues 189–358 and 371–465), suggesting that VNT-101 inhibits the homo-oligomerization of NP to exert antiviral activity.

**Fig 1:**
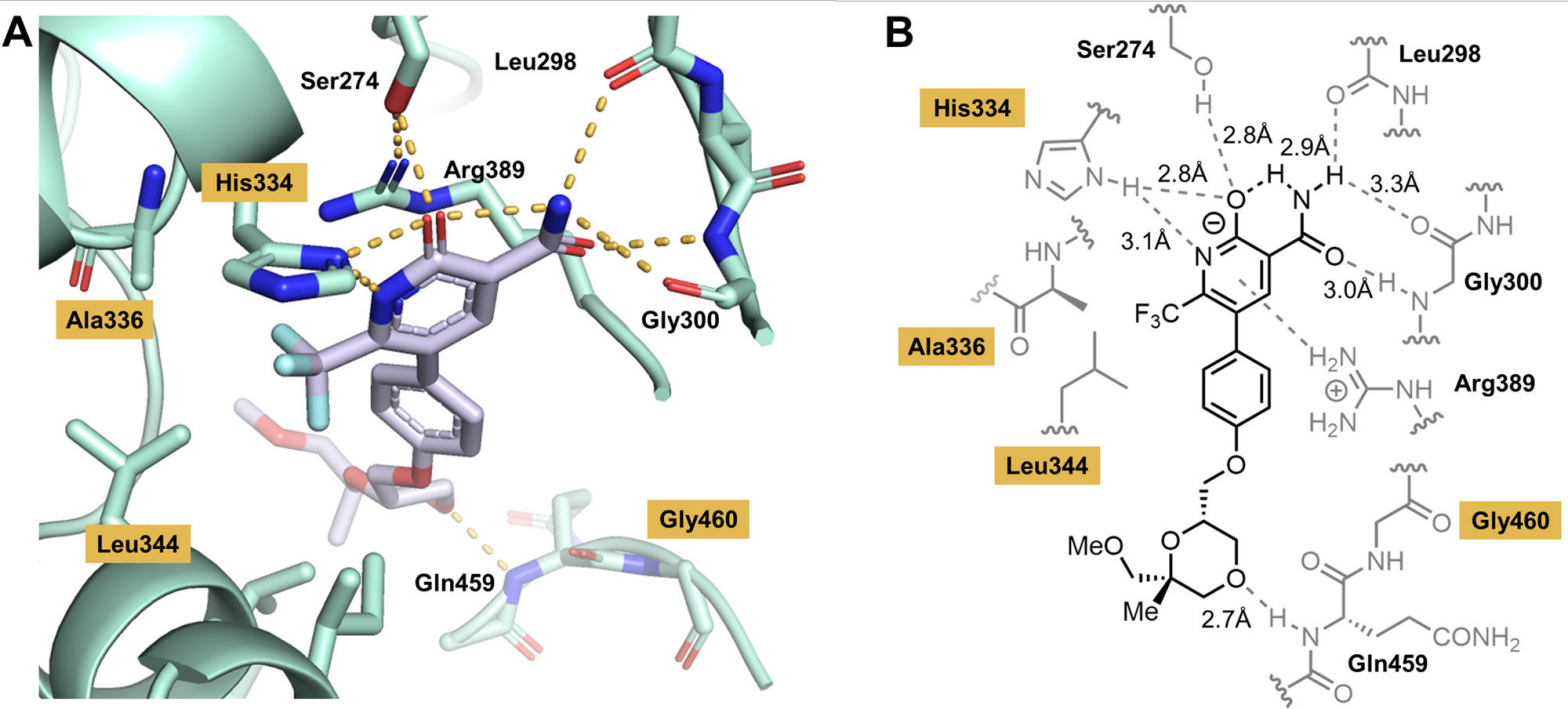
Co-structure of VNT-101 complexed with influenza nucleoprotein. (**A**) VNT-101 in the binding pocket of NP, with key residues noted. (**B**) Structure of VNT-101 and schematic showing NP residues involved in binding interaction in gray. Hydrogen bond distances are shown in Å. In both (**A**) and (**B**), yellow highlighting indicates amino acid positions on NP where resistance-associated variants were identified in the *in vitro* selection study. Abbreviations: NP, nucleoprotein.

To further validate the mechanism of action of VNT-101, the co-structure of VNT-101 complexed with recombinant NP from influenza A/Puerto Rico/8/1934 was solved by X-ray crystallography, allowing visualization of the key binding interactions with the target protein (Fig. 1). VNT-101 engages in several polar interactions with residues lining the binding pocket of the protein. The pyridine ring is negatively charged at physiological pH and forms a salt bridge with histidine 334 (HIS-334), with both the ring nitrogen and carbonyl oxygen atoms approaching within 3.2 Å. Additionally, a hydrogen bond is formed between the pyridine carbonyl and the hydroxyl group of serine 274 (SER-274). The anionic pyridone ring also interacts with cationic arginine 389 (ARG-389) through both cation-π and electrostatic interactions, further stabilizing this ring in the binding pocket. The amide substituent of VNT-101 forms hydrogen-bonding interactions with the backbone carbonyl of leucine 298 and the backbone amine of glycine 300 (GLY-300). The CF_3_ substituent plays a key role in lowering the pKa of VNT-101 and is 3.4 Å from a methyl group of leucine 344. The steric bulk of the CF_3_ group also forces the central phenyl ring of VNT-101 to twist out of plane with the pyridone ring, as illustrated in the binding mode of the ligand. The ether linker from the phenyl ring orients the methylene group planar to the phenyl substituent, placing the dioxane such that the 4-oxygen of the dioxane substitute of VNT-101 interacts with the backbone amine of glutamine 459 (GLN-459). The quaternary substituent at the 2-position of the dioxane fills the lipophilic region of the binding pocket, displacing a water molecule observed in related analogs, and also reducing the flexibility of the dioxane ring, thereby lowering the entropic penalty upon binding of the ligand.

The four mutant residues associated with resistance in the *in vitro* selection studies (H334, A336, L344, G460) are highlighted in Fig. 1. In all cases, the side chains are pointed toward the ligand. The H334Y mutation would remove the salt bridge partner for the key interaction with the anionic pyridone ring. Mutations A336T and L334S introduce polarity to a region occupied by the lipophilic CF_3_ group. In the case of the G460R mutant, the introduction of a larger, polar residue in place of glycine likely impacts the ability of the dioxane tail to bind and/or establish the key hydrogen-bonding interaction with the backbone NH of GLN-459. In all cases, these variants would directly alter the interaction of the ligand with the binding pocket and reduce its affinity.

These data demonstrate that VNT-101 binds to influenza NP in the oligomerization binding pocket, strongly implying that the drug disrupts homo-oligomerization of NP by steric occlusion, thereby inhibiting viral replication. This inference is further corroborated by biochemical experiments that were conducted using VNT-725, an early-stage analog of VNT-101. When VNT-725 was added in increasing amounts to a preparation of recombinant, predominantly trimeric NP, gel filtration analysis revealed a pronounced, dose-dependent shift toward the accumulation of NP monomers, with a corresponding decrease in the oligomer population (Fig. S1).

### VNT-101 has potent and specific activity against influenza A virus

The activity of VNT-101 against different influenza virus isolates was evaluated by CPE assays in MDCK cells. A total of 10 influenza A H1N1 isolates, six influenza A H3N2 isolates, and two influenza B isolates were evaluated. Overall, VNT-101 showed potent inhibitory activity against nine of the H1N1 isolates and five of the H3N2 isolates, with mean EC_50_ values ranging from 1.85 to 122.57 nM (Table 2). No activity was detected against the laboratory strains A/Malaya/302/1954 (H1N1) or A/Hongkong/8/1968 (H3N2) up to a top concentration of 50 µM. Analysis of the NP sequences for these two isolates revealed the presence of a variant in NP at position 334, H334N. This likely results in a similar loss of the salt bridge interaction with VNT-101, which is implicated in the H334Y mutant identified as a high-level resistance-associated variant (RAV) in the *in vitro* resistance selection study (Table 1). It is notable that these two isolates, as well as several others with lower susceptibility to VNT-101 (e.g., A/Weiss/1943, A/WS/1933, and A/WSN/1933), were isolated 50–90 years ago and represent laboratory-adapted isolates. Focusing on the more recently circulating clinical isolates (i.e., those identified since the 2009 pandemic), VNT-101 showed potent inhibitory activity against all H1N1 and H3N2 isolates tested, with mean EC_50_ values ranging from 1.85 to 17.68 nM (Table 2; strains in bold text). Importantly, no significant activity was detected against influenza B viruses (Table 2).

**TABLE 2:** Antiviral activity of VNT-101 against IAV H1N1 and H3N2 isolates and influenza B viruses^*a*^

Virus strain	Subtype	Year of isolation	VNT-101 (nM)			
			EC_50_	SD	EC_90_	SD
**A/Brisbane/05/2018**	H1N1	2018	6.55	2.33	11.24	2.01
**A/Michigan/45/2015**	H1N1	2015	17.68	8.98	29.43	9.07
**A/California/07/2009**	H1N1	2009	8.39	0.31	19.20	2.74
**A/Mexico/4108/2009**	H1N1	2009	14.41	4.77	112.20	151.45
**A/New York/18/2009**	H1N1	2009	1.85	0.73	5.12	4.83
A/Malaya/302/1954	H1N1	1954	>50,000	n/a	>50,000	n/a
A/Weiss/1943	H1N1	1943	122.57	27.80	209.78	77.01
A/Puerto Rico/8/1934	H1N1	1934	2.08	0.01	3.22	0.02
A/Wilson-Smith/1933	H1N1	1933	26.26	10.67	50.64	22.17
A/WSN/1933	H1N1	1933	114.93	48.76	191.00	94.20
**A/Kansas/14/2017**	H3N2	2017	3.14	2.10	17.96	20.61
**A/Hong Kong/4801/2014**	H3N2	2014	2.59	1.62	3.71	2.59
**A/California/2/2014**	H3N2	2014	2.20	0.17	3.41	0.27
**A/Switzerland/971593/2013**	H3N2	2013	2.19	0.84	3.32	1.65
**A/Perth/16/2009**	H3N2	2009	4.05	1.93	5.68	2.76
A/Hong Kong/8/1968	H3N2	1968	>50,000	n/a	>50,000	n/a
**B/Florida/78/2015**	n/a	2015	43,670.00	6,261.94	>50,000	n/a
B/Lee/1940	n/a	1940	>47,500	n/a	>50,000	n/a

^*a*^ VNT-101 activity was tested using a CPE-based assay on MDCK cells; data are expressed as the mean EC_50_ and SD from three independent experiments. Bold text indicates data from most recent (2009–2018) and more relevant clinical isolates of influenza. n/a, not applicable; SD, standard deviation.

Although IAVs are known to infect a broad range of animals, their ultimate reservoir is among wild waterfowl species, which host an enormous variety of viral strains, most of which have relatively low pathogenicity. Some strains, however, are highly virulent. These so-called highly pathogenic avian influenza (HPAI) isolates are of great concern because, when transmitted to humans, they induce a severe, systemic disease of high lethality. Transmission to humans is infrequent and typically occurs on poultry farms, due to high bird densities and prolonged, close contact with farmers. Fortunately, efficient human-to-human spread has not yet been observed, but the possibility that HPAI isolates could incorporate changes that promote such transmission makes these strains of great concern to public health authorities. For this reason, we tested a limited set of HPAI isolates (three H5N1 and two H7N9) that have transmitted to humans, including isolate A/cattle/Texas/56283/2024 (H5N1), which is representative of the clade 2.3.4.4b isolates currently circulating in wild waterfowl in the USA and Europe (with sporadic spread to commercial poultry flocks in these locations), as well as in dairy cattle in the USA. VNT-101 was active against all five tested isolates, with EC_50_ values ranging from 19 to 69 nM (Table 3). Although more extensive surveys of HPAI strains will be required, the activity against HPAI isolates recovered from humans suggests that VNT-101 could be useful in the event of an outbreak of human-adapted HPAI viruses.

**TABLE 3:** Antiviral activity of VNT-101 against HPAI H5N1 and H7N9 isolates^*a*^

Virus strain	Subtype	Source	VNT-101 (µM)	
			EC_50_	CC_50_
A/Vietnam/1203/2004	H5N1	Human	0.025	>10
A/Hong Kong/213/2003	H5N1	Human	0.019	>10
A/cattle/Texas/56283/2024	H5N1	Cattle	0.065	>30^*b*^
A/Taiwan/1/2017	H7N9	Human	0.069	>10
A/Anhui/1/2013	H7N9	Human	0.024	>10

^*a*^ VNT-101 activity was tested using a CPE-based assay on MDCK cells (*n *= 1). HPAI, highly pathogenic avian influenza.

^*b*^ CC_50_ > 30 µM generated in an independent experiment on MDCK cells.

### VNT-101 has a favorable cytotoxicity profile

The cytotoxicity of VNT-101 was evaluated across five cell lines under conditions in which cell proliferation occurred: MDCK (canine kidney cell line), A549 (human lung carcinoma cell line), HepG2 (human hepatocarcinoma cell line), HEK293 (human embryonic kidney cell line), and MT-4 (human CD4 + T cell line). VNT-101 showed no cytotoxicity on MDCK, A549, and HepG2 cells up to 100 µM, the highest concentration tested, and generated CC_50_ values of 72.83 µM on HEK293 and 54.05 µM in MT-4 cells (Table 4). Three of these cell lines (A549, HepG2, HEK293) were selected to evaluate VNT-101-induced cytotoxicity under stationary (non-dividing) conditions. VNT-101 showed no cytotoxicity on A549 and HepG2 cells under stationary conditions up to 100 µM, the highest concentration tested, and generated CC_50_ values on two of the three independent runs on HEK293 cells (CC_50_ = 68.17 µM, 72.89 µM, >100 µM; average >80.35 µM; Table 4). Selectivity indices (SI) were calculated for each cell line using the mean CC_50_ value from the most sensitive conditions for cytotoxicity (proliferating conditions) and the mean EC_50_ value from the least susceptible recently circulating IAV isolate tested (A/Michigan/45/2015, EC_50_ = 17.68 nM). The resulting SI values for VNT-101 were >3,000 for the five cell lines tested, demonstrating a favorable cytotoxicity profile.

**TABLE 4:** Cytotoxicity and selectivity indices of VNT-101^*a*^

Cell line, source	Proliferating condition CC_50_ (μM)	Stationary condition CC_50_ (μM)	Selectivity index
MDCK, canine kidney	>100	Not tested	>5,656
A549, human lung	>100	>100	>5,656
HepG2, human liver	>100	>100	>5,656
HEK293, human kidney	72.83	>80.35	4,119
MT-4, human T cell	54.05	Not tested	3,057

^*a*^ Cytotoxicity data are presented as the mean CC_50 _from three independent experiments. Selectivity index is calculated as the ratio of the mean CC_50 _value (proliferating conditions) over the mean EC_50_ value for A/Michigan/45/2015, the least susceptible currently circulating (2009 to present) influenza A strain tested (EC_50_ = 0.01768 μM).

### No cross-resistance detected between VNT-101, baloxavir, and oseltamivir

Of the four mutations in NP identified with *in vitro* resistance selection studies (H334Y, A336T, L344S, G460R), the L344S mutation had a smaller impact on the EC_50_ in the mini-genome reporter assay (13.6-fold shift, compared with >1,428-fold shift for H334Y, A336T, and 48.3-fold shift for G460R; Table 1). In addition, VNT-101 has shown potent activity against multiple isolates with the NP L344S mutation in the CPE assay (e.g., A/New York/18/2009, A/California/07/2009, A/Mexico/4108/2009), suggesting that this may not be a critical RAV. The three mutations confirmed as RAVs for VNT-101 were tested for cross-resistance to baloxavir in the mini-genome reporter assay (Table 5). Similar to previous results (Table 1), the H334Y and A336T mutations caused the maximum shift in VNT-101 EC_50_ allowed by the concentration testing range (>941-fold shift in EC_50_), while the G460R mutation resulted in a 16.42-fold shift in VNT-101 EC_50_, relative to the wild-type construct. In contrast, all three RAVs remained fully susceptible to inhibition with baloxavir, with EC_50_ values ranging from 1.75 to 2.40 nM (compared to wild-type EC_50_ = 2.56 nM; <1-fold shift for each). Direct testing for cross-resistance to oseltamivir in the mini-genome reporter assay is not possible, since the target of oseltamivir, neuraminidase, is not present in this assay format. However, two IAV isolates containing the NP H334N RAV (VNT-101 EC_50_ >50 µM) were tested against oseltamivir and baloxavir in the CPE assay format. Both oseltamivir and baloxavir remained highly potent against these two isolates, with a <2-fold shift in EC_50_ values relative to a designated reference strain (A/Puerto Rico/8/1934 [H1N1]), which harbors none of the VNT-101 RAVs.

**TABLE 5:** Testing for cross-resistance between RAVs of VNT-101, baloxavir, and oseltamivir^*e*^

Virus strain	Mutation	VNT-101		Baloxavir		Oseltamivir^*a*^	
		EC_50_ (μM)	Fold shift	EC_50_ (nM)	Fold shift	EC_50_ (μM)	Fold shift
VNT-101 RAVs							
A/Puerto Rico/8/1934^*b*^	Reference	0.011	n/a	2.56	n/a		
A/Puerto Rico/8/1934^*b*^	NP H334Y	>10	>941	2.40	0.94		
A/Puerto Rico/8/1934^*b*^	NP A336T	>10	>941	2.24	0.87		
A/Puerto Rico/8/1934^*b*^	NP G460R	0.18	16.42	1.75	0.71		
A/Puerto Rico/8/1934^*c*^^,*d*^	Reference	0.002	n/a	0.57	n/a	0.97	n/a
A/Malaya/302/1954^*c*^^,^^*d*^	NP L344S, NP H334N	>50	>25,000	1.10	1.93	1.15	1.19
A/Hong Kong/8/1968^*c*,*d*^	NP H334N	>50	>25,000	0.68	1.19	0.09	0.09
Baloxavir RAV							
A/Puerto Rico/8/1934^*c*^	Reference	0.0021	n/a	0.57	n/a	0.97	n/a
Baloxavir-resistant A/Puerto Rico/8/1934^*c*^	PA I38T	0.0021	0.99	104.73	183.74	2.81	2.89
Oseltamivir RAV							
A/Weiss/1943^*c*^	Reference	0.123	n/a	0.40	n/a	1.05	n/a
Oseltamivir-resistant A/Weiss/1943^*c*^	NA H275Y	0.118	0.96	0.42	1.05	>78.39	>74.66

^*a*^ Activity with oseltamivir is not expected in the mini-genome reporter assay because the neuraminidase target is not present.

^*b*^ EC_50_ values were generated using the mini-genome reporter assay; data are expressed as the mean EC_50_ from three independent experiments. Fold shift is calculated as the ratio of the EC_50_ for the mutant NP construct / the EC_50_ for the reference NP construct.

^*c*^ EC_50_ values were generated using a CPE-based assay on MDCK cells; data are expressed as the mean EC_50_ from three independent experiments. Fold shift is calculated as the ratio of the EC_50_ for the isolate with NP mutations / the EC_50_ for the designated reference isolate.

^*d*^ A/Puerto Rico/8/1934 (H1N1) is designated as the reference isolate for VNT-101 for the purposes of calculating fold shift for the CPE assay because all three compounds show potent activity against this isolate and there are no NP mutations present that are associated with reduced susceptibility to VNT-101.

^*e*^ CPE, cytopathic effect; NA, influenza neuraminidase; NP, influenza nucleoprotein; PA, polymerase acidic protein (endonuclease); RAV, resistance-associated variant. n/a indicates that a fold-shift value is not applicable. The A/Puerto Rico/1934 strain serves as the reference strain against which fold-shift values for other isolates are calculated. Likewise, A/Weiss/1943 is the reference for determining the fold shift of the oseltamivir-resistant derivative.

Similarly, IAV isolates with mutations conferring resistance to baloxavir (PA I38T) or oseltamivir (NA H275Y) were tested for cross-resistance to VNT-101 using the CPE assay (Table 5). VNT-101 showed similar activity against the drug-resistant and wild-type (reference) influenza isolates, with <1-fold shift in EC_50_ values. Taken together, these data indicate that there is no cross-resistance between RAVs of VNT-101, baloxavir, and oseltamivir.

### VNT-101 is highly effective in a lethal influenza challenge model in mice

Intranasal infection of mice with high doses of IAV induces a rapidly lethal pneumonia. The ability of antiviral drugs to rescue mice from this lethality is a standard preclinical assay for *in vivo* antiviral activity (23, 24). In experiments reported in detail elsewhere (15), we demonstrated that VNT-101, when administered simultaneously with the IAV inoculum (i.e., as prophylaxis), protected mice from lethality (for the convenience of the reader, these studies are summarized in Fig. S2). Optimal protection from infection (superior to that afforded by oseltamivir) was achieved by dosing 30 mg/kg VNT-101 PO twice daily (BID) for 5 days. At this maximally effective dose, the unbound plasma C_min_ for VNT-101 was approximately 20-fold over the *in vitro* EC_50_ value (Table S1).

**Fig 2:**
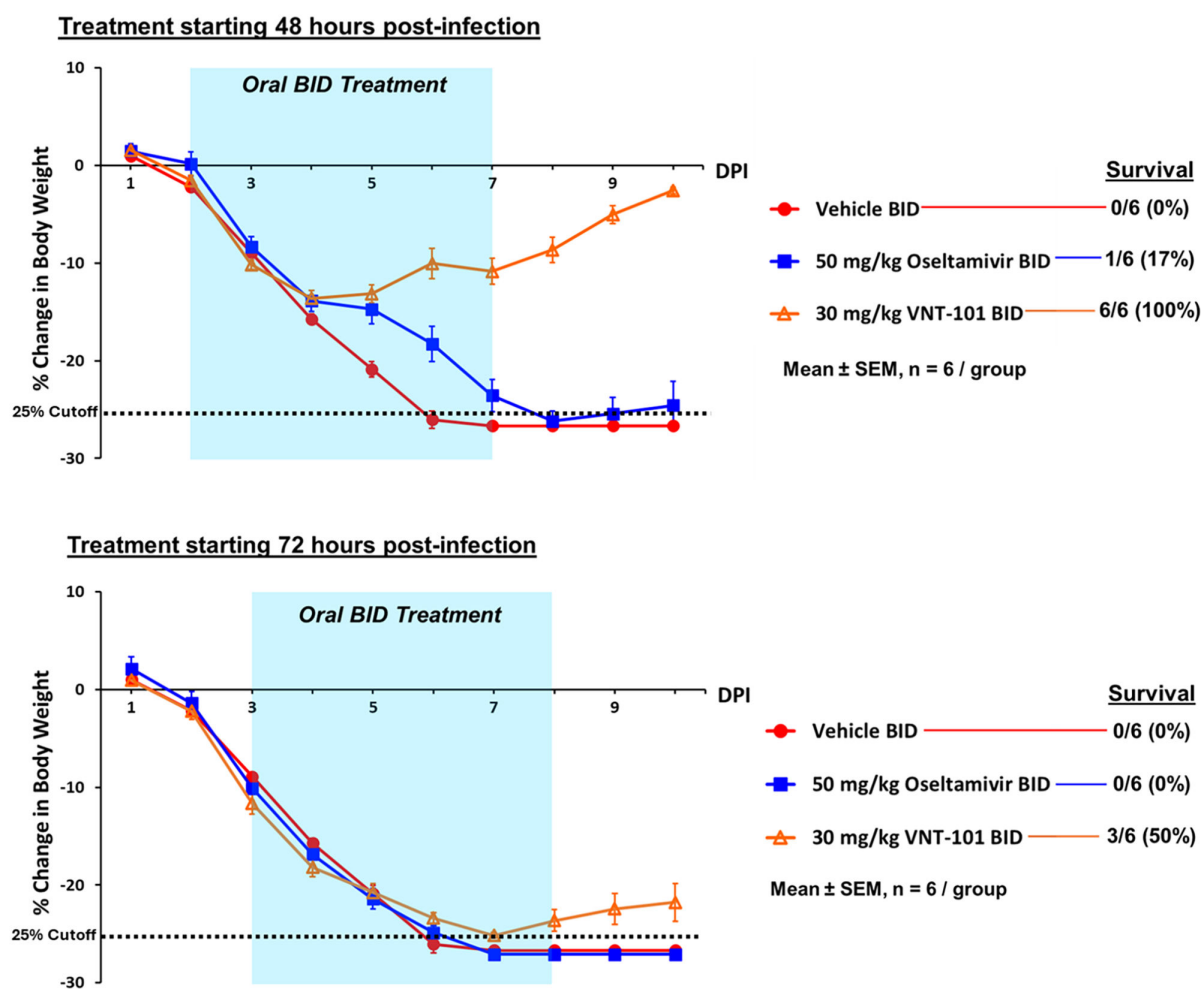
Change in body weight with VNT-101 delayed treatment. Percent change in body weight is calculated based on initial measurement taken on day 0, with the last body weight observation carried forward. The X-axis indicates days post-infection up to the end of the study (day 10). The blue highlighted area indicates duration of treatment with VNT-101 or oseltamivir (total of 5 days). A dotted line denotes 25% body weight loss cutoff, at which point animals were euthanized in accordance with ethical guidelines. Abbreviations: DPI, days post-infection; BID, twice a day; SEM, standard error of the mean.

Accordingly, this dose was selected for further studies of the effectiveness of VNT-101 in the therapeutic context (i.e., administration of drug starting 48 or 72 h post-inoculation with IAV). Mice were inoculated intranasally with 2 × LD_100_ of IAV strain A/Puerto Rico/8/1934 (H1N1), followed by PO administration of 30 mg/kg VNT-101 or 50 mg/kg oseltamivir BID for a total of 5 days, starting 48 or 72 h post-infection (hpi). In the vehicle control group, all six animals experienced consistent weight loss over time, resulting in >25% body weight loss and euthanasia (consistent with Novartis’ ethical guidelines) by day 6 or 7 (Fig. 2; Fig. S3). Administration of VNT-101 initiated at 48 hpi resulted in survival of all six animals to the end of the study, with a maximum mean body weight loss of 13.6% on day 4, followed by a gradual recovery to a mean body weight loss of 2.6% on day 10. In contrast, only one animal survived in the parallel group treated with oseltamivir. Delaying the start of VNT-101 administration until 72 hpi resulted in only three of six animals surviving to the end of the study, with a maximum mean body weight loss of 25.1% on day 7, followed by a modest recovery to a mean body weight loss of 21.8% on day 10, driven by the three surviving animals. In contrast, no animals survived in the parallel group treated with oseltamivir.

### Combinations of VNT-101 plus oseltamivir improve survival with 72-hour delayed treatment

**Fig 3:**
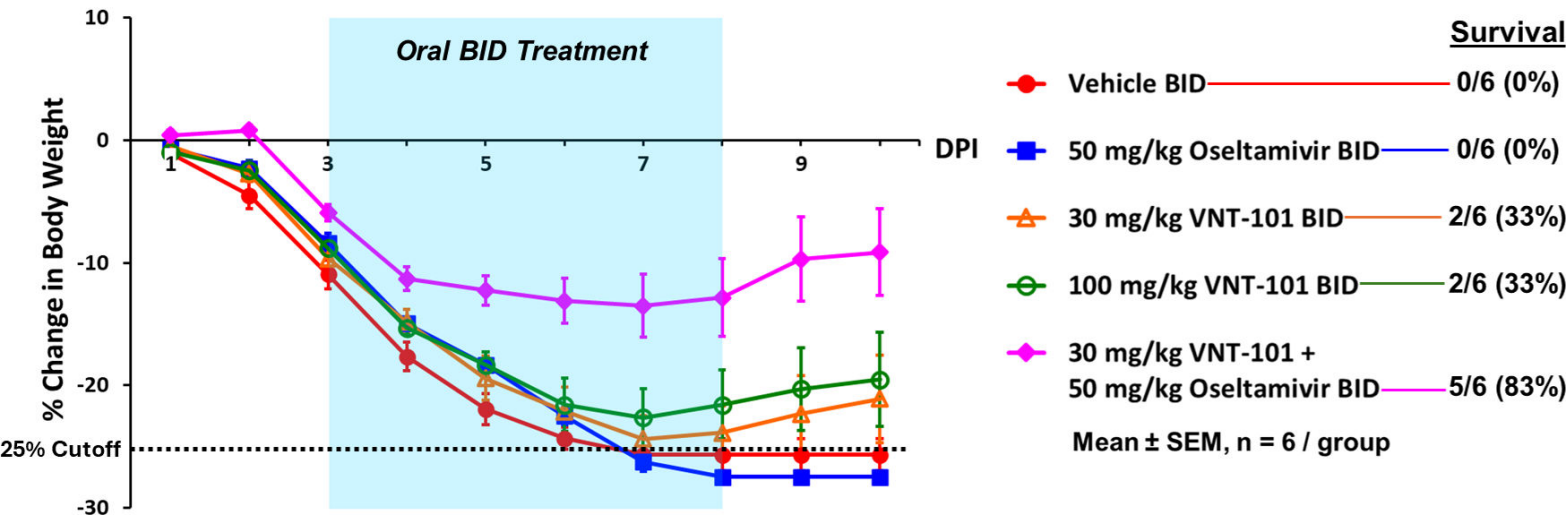
Change in body weight with a combination of VNT-101 plus oseltamivir. Percent change in body weight (Groups 1, 3, 5, 7, and 9 [*n* = 6 per group]) is calculated based on initial measurement taken on day 0, with the last body weight observation carried forward. The X-axis indicates days post-infection up to the end of the study (day 10). The blue highlighted area indicates the duration of treatment with VNT-101 or oseltamivir (total of 5 days). A dotted line denotes 25% body weight loss cutoff, at which point animals were euthanized in accordance with ethical guidelines. Abbreviations: DPI, days post-infection; BID, twice a day; SEM, standard error of the mean.

In a final study, we investigated whether the survival results obtained with 72-hour delayed treatment with VNT-101 alone might be improved by a combination therapy of VNT-101 plus oseltamivir. Mice were inoculated intranasally with 2 × LD_100_ of IAV, followed by PO administration of 30 mg/kg or 100 mg/kg VNT-101 BID alone, 50 mg/kg oseltamivir BID alone, or a combination of 30 mg/kg VNT-101 and 50 mg/kg oseltamivir BID for a total of 5 days, starting at 72 hpi. All six animals in the vehicle control group and the oseltamivir-treated group experienced consistent weight loss over time, resulting in >25% body weight loss and euthanasia by day 7 or 8, respectively (Fig. 3; Fig. S4). Treating with 30 mg/kg VNT-101 BID resulted in a maximum mean body weight loss of 24.4% on day 7. Only two of six animals survived to day 10, with a final mean body weight loss of 21.1%, similar to previous observations (Fig. 2; Fig. S3). Increasing the dose of VNT-101 to 100 mg/kg BID did not improve survival over the 30 mg/kg BID dose. In contrast, animals treated with a combination of 30 mg/kg VNT-101 plus 50 mg/kg oseltamivir BID showed marked improvements in body weight loss and survival, with five of six animals surviving to day 10, a maximum mean body weight loss of 13.5% on day 7, and recovery to a final mean body weight loss of 9.1%.

**Fig 4:**
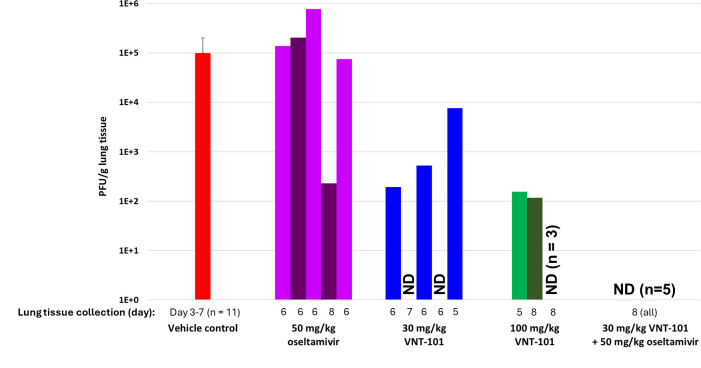
Lung viral loads with a combination of VNT-101 plus oseltamivir. Lung tissue was collected for viral load quantitation either at the end of the treatment phase (day 8) or at the time of death or euthanasia for moribund animals (if prior to day 8). Each bar represents the lung viral load (PFU/g) for an individual animal in the indicated dose group. Animals with no detectable virus in the lung homogenate are indicated with “ND” for not detected. For vehicle control (Groups 1 and 2), viral load data from individual animals (*n* = 11) are averaged and presented as a single value ± standard deviation. The day on which lung tissue was collected for individual animals or across the group is shown below the X-axis on the graph. Abbreviations: PFU/g; plaque-forming unit per gram of lung tissue; ND, not detected.

We also examined the infectious viral load (in PFU/g of tissue) in the lungs of the animals in each dosing cohort either at the end of the treatment phase (day 8) or at the time of death or euthanasia for moribund animals (Fig. 4). Lung viral loads in animals treated with 50 mg/kg oseltamivir BID were comparable to the vehicle control (10^5^ PFU/g average lung viral load in all untreated or vehicle control animals; *n* = 11). Treating with 30 mg/kg or 100 mg/kg VNT-101 BID resulted in two or three animals, respectively, with no detectable virus in the lung tissue homogenate. The remaining animals in each group had viral loads that were lower than those seen after oseltamivir treatment (with the exception of one outlier animal in the oseltamivir group with lower titers). In contrast, none of the animals treated with the combination of 30 mg/kg VNT-101 plus 50 mg/kg oseltamivir BID had detectable viral loads in the lung tissue homogenate.

These data demonstrate that dosing with the combination of 30 mg/kg VNT-101 plus 50 mg/kg oseltamivir BID for 5 days, starting at 72 hpi, showed meaningful improvement (83% survival, no detectable lung viral load) compared with either drug alone, suggesting a clear benefit of combination therapy in this context.

## DISCUSSION

This study reports the characterization of VNT-101, a novel inhibitor of influenza A virus, which was optimized from a hit discovered by phenotypic screening of a diverse chemical library, followed by extensive medicinal chemistry optimization (15). VNT-101 is highly specific for IAV: no activity was observed against IBV. The drug has potent activity against currently circulating strains of seasonal IAV and against HPAI isolates that have been successfully transmitted to humans. VNT-101 targets the oligomerization domain of the viral NP and thereby disrupts NP oligomerization, which is essential for the formation of an RNP capable of supporting correct viral RNA synthesis (5). As expected, given that this mechanism of action is distinct from those of oseltamivir and baloxavir, VNT-101 remains active against mutants resistant to these currently approved drugs.

We directly tested a small collection of IAV strains for susceptibility to VNT-101 in culture. All of those isolated in the past 15 years were sensitive to the drug (Table 2). To examine susceptibility more systematically, we examined the frequency of the RAVs identified in the *in vitro* resistance selection studies and confirmed in the minigenome assay (H334Y, A336T, L344S, G460R) in influenza NP sequences reported in the National Institute of Allergy and Infectious Diseases Influenza Research Database (25). The analysis focused on NP sequences isolated from human sources with a collection date of 2009 to present (to represent currently circulating, highly relevant strains) from IAV H1N1, H3N2, H5N1, and H7N9 subtypes. H334Y was not observed in any reported sequences included in this analysis; however, the H334N mutation was present at 2.32% frequency in H1N1 strains only. When strains isolated prior to 2009 were examined, residue 334 fluctuated over the years between histidine and asparagine within both H1N1 and H3N2 subtypes. This fact suggests that mutations at residue 334 can be viable and retain a degree of fitness. We reiterate, however, that histidine at residue 334 predominates (≥97.66%) in currently circulating strains of both H1N1 and H3N2, and, in fact, H334 has prevailed in H3N2 strains for the last 42 years. There is no significant frequency (≤0.86%) of A336T or G460R (or any other substitution at those positions) reported in the database across IAV subtypes. Finally, the RAV S344 predominates (≥99.94%) in NP sequences from the H1N1, H5N1, and H7N9 subtypes; however, this mutation caused only a modest shift in potency in the mini-genome assay (13.6-fold shift in EC_50_; Table 1), and strains with the L344S mutation were still potently inhibited by VNT-101 in the CPE-based assay (EC_50_ ranging from 1.85 nM to 122.57 nM for seven H1N1 strains with only the L344S mutation; Table 2), suggesting that L344S is not a primary driver for reduced susceptibility to VNT-101 in the context of viral infection.

Small molecules that are able to efficiently block protein-protein interactions are not very common, despite intense pharmaceutical industry interest in such inhibitors. This is likely because most protein-protein interactions occur across surfaces that are relatively large, flat, and hydrophobic, devoid of well-defined pockets or clefts suitable for binding small ligands with high affinity (26). However, influenza virus NP oligomerization does not follow this pattern—it is based on insertion of a structured loop from one NP monomer into a discrete pocket in the body of a nearby monomer. It is interesting to note that in the very first description of the NP crystal structure (18), the authors of that study recognized the potential druggability of this interaction: “We anticipate that chemical compounds which competitively displace the tail loop from its binding pocket would interfere with viral genome replication, and therefore serve as promising leads for anti-influenza drug development.” Our discovery of VNT-101 serves as a striking validation of this prediction.

Both genetic and biochemical experiments reveal that the mechanism of action of VNT-101 is distinct from that of nucleozin and its analogs (which have also been shown to target NP). First, IAV mutants that are highly resistant to VNT-101 retain full susceptibility to nucleozin (Table 1). While we have not selected nucleozin-resistant mutants, those described in earlier studies (e.g. Y289H, N309T) (17, 27, 28) do not correlate with residues we have identified as critical for VNT-101 function. Second, the biochemical phenotype of the drugs is radically different: nucleozin induces aberrant oligomerization (aggregation) of NP, while VNT-101 disrupts NP oligomer formation. Finally, examination of the co-structures of VNT-101 or nucleozin bound to NP reveals that they bind to different sites on the protein: one molecule of nucleozin interacts with two sites (the Y289/N309 pocket and the R382 pocket [27]) located on different NP trimers, and this interaction crosslinks the trimers, resulting in higher-order oligomers.

The medicinal chemistry effort that resulted in VNT-101 optimized the compound for antiviral potency as well as other important pharmacokinetic properties, including oral bioavailability and clearance (15). These properties allowed VNT-101 to perform well in a mouse model of lethal IAV infection. This model produces a rapidly progressive pulmonary infection with high lethality. As such, it is representative of a more severe infection than that seen in typical seasonal influenza and therefore serves as a stringent test of the *in vivo* antiviral effect of VNT-101. In this regard, it is encouraging that 30 mg/kg VNT-101 BID outperformed 50 mg/kg oseltamivir BID in both prophylaxis and therapeutic experimental designs. We emphasize that we do not interpret these data to imply that VNT-101 will prove superior to oseltamivir in clinical trials in seasonal influenza. In this context, we note that baloxavir also outperformed oseltamivir in the mouse model of IAV infection but has not proven superior in resolving clinical symptoms in human seasonal influenza (11, 29). Rather, we view the result as simply indicating that the *in vivo* efficacy of the compound is more than sufficient to warrant continued development for human therapeutic use. In addition, based on these experiments, we propose that maintenance of free (non-protein bound) drug levels an order of magnitude over the *in vitro* EC_50_ value throughout the dosing interval may be a useful, if conservative and provisional, target for optimizing human therapy with VNT-101.

Although we are encouraged by the increase in efficacy observed in our limited studies with combinations of VNT-101 and oseltamivir, we do not necessarily expect similar results in typical seasonal influenza infections in humans, where the infection is milder than that established in the lethal influenza challenge model in mice. Previous studies of combination therapy in seasonal human influenza have generally not shown dramatic effects on abbreviation of clinical symptoms (30, 31). We speculate, however, that there may be particular situations in which such combinations might have value—for example, in rapidly progressive, potentially lethal human infections with HPAI strains, or in infections of immunocompromised hosts. In the latter case, viral replication is often prolonged, with the evolution of multiple variants; combination therapy in such cases might abbreviate the period of active genomic replication and thus reduce the risk of evolution of mutants resistant to either drug. These and other scenarios for use of VNT-101 in combination therapy will ultimately need to be tested in clinical trials, planning for which is underway.

## MATERIALS AND METHODS

### Cell lines and compounds

Madin-Darby canine kidney (MDCK) epithelial cell line (American Type Culture Collection [ATCC] CCL-34) was cultured in Eagle’s Minimum Essential Medium (EMEM) supplemented with 10% fetal bovine serum (FBS). Human embryonic kidney epithelial cell line (HEK293; ATCC CRL-1573) and an HEK293 cell line with constitutive expression of SV40 T-antigen (293T, ATCC CRL-3216; HEK293T/17, ATCC CRL-11268) were cultured in Dulbecco’s Modified Eagle Medium (DMEM) supplemented with 10% FBS. Human lung carcinoma cell line (A549; ATCC CCL-185) and human hepatocarcinoma cell line (HepG2; ATCC HB-8065) were cultured in RPMI 1640 supplemented with 10% FBS. Human CD4 + T cell line (MT-4) was obtained from the Wuhan Institute of Virology, and cells were cultured in RPMI 1640 supplemented with 10% FBS. VNT-101 was synthesized in-house at Novartis or at Sai Life Sciences (Telangana, India). VNT-725 was synthesized in-house at Novartis. Nucleozin (CAS No. 341001-38-5) and oseltamivir (CAS No. 204255-11-8) were obtained from Sigma-Aldrich (St. Louis, MO, USA).

### Influenza neuraminidase assay-based screen

MDCK cells were seeded at 2,000 cells/well in 1,536-well plates in neuraminidase (NA) assay medium (RPMI without phenol red, 0.1% BSA, 1% penicillin-streptomycin) and allowed to adhere overnight. The next day, compound solution was added to cells, followed by infection with H1N1 influenza virus A/Puerto Rico/8/1934 (ATCC VR-95) at a multiplicity of infection (MOI) of 0.01 PFU/cell, diluted in NA assay medium containing L-(tosylamido-2-phenyl) ethyl chloromethyl ketone (TPCK)-treated trypsin at a final concentration of 0.5 µg/mL. After incubation for 48 h at 37°C and 5% CO_2_, the level of infection was quantitated using the NA-XTD Influenza Neuraminidase Assay Kit (Applied Biosystems, cat #4457535), according to the manufacturer’s instructions. NA activity was determined as a percentage of virus control (DMSO-treated, virus-infected cells).

To determine cytotoxicity, cell viability was measured in parallel in compound-treated, uninfected MDCK cells using CellTiter-Glo Luminescent Cell Viability Assay (Promega, cat #G7573) according to the manufacturer’s instructions.

### Influenza cytopathic effect (CPE) assay

MDCK cells were plated at a density of 1 × 10^4^ cells/well (384-well plate) or 1.5–3.0 × 10^4^ cells/well (96-well plate) and cultured overnight at 37°C and 5% CO_2_. The next day, serially diluted compound solution and virus, diluted in assay medium (EMEM, MEM, DMEM, or OptiPRO SFM supplemented with 1–10 µg/mL TPCK-treated trypsin) to achieve an MOI ranging from 0.0005 to 0.012 PFU/cell for influenza A H1N1 and H3N2 isolates, from 0.0005 to 0.5 PFU/cell for H5N1 and H7N9 isolates, and from 0.006 to 0.159 PFU/cell for influenza B isolates, were added to the cells, and plates were incubated for 3–5 days at 37°C and 5% CO_2_. Following incubation, cell viability (i.e., protection of cells from CPE induced by viral infection) was measured using (i) CellTiter-Glo Luminescent Cell Viability Assay according to the manufacturer’s instruction, (ii) Cell Counting Kit 8 (WST-8/CCK8; Shanghai Life iLAB Technology, cat #AC11L057) according to the manufacturer’s instruction, or (iii) neutral red dye (stain cells for 2 h, wash with PBS, extract incorporated dye in 50:50 Sorensen citrate buffer:ethanol for >30 min, and measure optical density at 540 nm). Readouts were determined as percent inhibition of virus-induced CPE relative to virus control (DMSO-treated, virus-infected cells) and cell control (DMSO-treated, uninfected cells). The concentration of the test compound required to inhibit CPE by 50% (EC_50_) or 90% (EC_90_) was reported.

To determine cytotoxicity, cell viability was measured in parallel in compound-treated, uninfected MDCK cells using the same conditions and methods as were used in the CPE assay. The concentration of the compound causing 50% cell death in the absence of virus (CC_50_) was reported.

### *In vitro* resistance selection studies

MDCK cells (plated at a density of 5–7.5 × 10^5^ cells/well in a 6-well plate the day before infection) were infected with influenza A virus strain A/Puerto Rico/8/1934 (H1N1) at an MOI of 0.01 PFU/cell in the presence of TPCK-treated trypsin (1 µg/mL) and increasing concentrations of VNT-101 (1×, 2×, 4× EC_50_) or DMSO as a control. Plates were examined daily for CPE (typically evident by 48–72 hpi), and the well with the highest concentration of VNT-101 and most extensive CPE (≥40%) was passaged (i.e., supernatant and cells were collected from the well and 100 µL of virus/cell material was used to infect freshly plated MDCK cells) at a higher concentration of VNT-101. If significant CPE was not evident, the concentration of VNT-101 remained the same as in the previous passage. Serial passaging continued through passage 6, where the VNT-101 concentration reached 5.12 µM (1,000× EC_50_). At each passage, the remaining virus/cell material from the passaged well was retained at −80°C to determine if mutations had developed as a result of selection with VNT-101 by multi-segment RT-PCR amplification, deep sequencing, and analysis. Additional details can be found in the Supplemental Methods.

### Mini-genome reporter assay (ribonucleoprotein [RNP] assay)

293T or HEK293T/17 cells were transfected with plasmids expressing NP, PA, PB1, and PB2 from A/Puerto Rico/8/1934 to form the RNP and an influenza A luciferase reporter plasmid (bearing IAV cis-acting sequences required for viral RNA synthesis) to readout replication activity. In addition, mutant NP constructs containing mutations (V194I, V270I, H334Y, A336T, L344S, and G460R) identified in H1N1 *in vitro* resistance selection studies were generated and used in place of the wild-type NP expression plasmid to evaluate the impact of each mutation on inhibition by VNT-101. Transfections were done in 96-well plates (5–6 × 10^4^ cells/well) or 384-well plates (1.8 × 10^4^ cells/well) using Fugene 6 transfection reagent (Promega, cat #E2692) for 293T cells and TransIT-293 transfection reagent (Mirus, cat #MIR2700) for HEK293T/17 cells, according to manufacturer’s instructions. Serially diluted compounds were added to cells 2 h post-transfection (293T cells) or 6 h post-transfection (HEK293T/17 cells), and the cells were incubated for a total of 48 h (293T cells) or 24 h (HEK293T/17 cells) at 37°C and 5% CO_2_.

Following incubation, cells were lysed, and intracellular luciferase expression was detected by Britelite Plus Reporter Gene Assay System (Perkin-Elmer, cat #6066769) or Dual-Luciferase Reporter Assay System (Promega, cat #E1960), according to the manufacturer’s instructions. To determine cytotoxicity (CC_50_), cell viability was measured in duplicate plates (but without plasmid transfection) using CellTiter-Glo Luminescent Cell Viability Assay according to manufacturer’s instructions.

### Co-structure determination by X-ray crystallography

NP from influenza A virus A/Puerto Rico/8/1934 (H1N1) (amino acids 8–498, His-tagged), at a concentration of 10 mg/mL in a buffer comprised of 20 mM Tris-HCl (pH 7.5), 200 mM NaCl, 10% glycerol (vol/vol), and 5 mM DTT, was mixed with 1 mM VNT-101. For crystallization trials set up with a 1:1 ratio, 1 µL NP protein-VNT-101 complex was mixed with 1 µL precipitant (0.1 M Tris [pH 8.5], 22.5% [wt/vol] PEG8000, and 0.1 M MgCl_2_) using the hanging drop method. Crystals grew at 4°C, and streak seeding was used to improve crystal quality. Crystals were harvested and cryopreserved in crystallization solution supplemented with 1 mM VNT-101 and 25% (vol/vol) ethylene glycol. The cryopreserved crystals diffracted to 2.33 Ǻ using synchrotron radiation. Data were processed using autoPROC (32) (Table S2). The co-structure was determined with molecular replacement using Phaser software (33) and a monomer of the trimeric native influenza NP (PDB Accession #2IQH) (18) as a search model. Refinement of the co-structure was completed by alternating rounds of real-space manual refitting of the model with *Coot* (34) and reciprocal-space refinement using the PHENIX (35) and BUSTER (36) programs. Co-structures were refined until convergence, and structural waters were added into suitable peaks in 2mF_o_-DF_c_ and mF_o_-DF_c_ maps at 1σ and 2σ levels, respectively.

### Cytotoxicity testing on stationary and proliferating cell lines

For proliferating conditions, 8-point, 3-fold serial dilution curves of compounds were prepared in DMSO and added to 96-well assay plates in triplicate wells. Cells were then seeded into compound-containing wells at 8,000 cells/well (HEK293, MT-4, A549, and HepG2) or 5,000 cells/well (MDCK) and cultured at 37°C and 5% CO_2_ for 3 days. For non-dividing conditions, cells were seeded in 96-well assay plates at 8,000 cells/well (HEK293, MT-4, A549, and HepG2) or 5,000 cells/well (MDCK) and cultured at 37°C and 5% CO_2_ overnight. Serial dilution curves (8-point, 3-fold) of compounds were then added to the assay plates in triplicate wells, and the cells were cultured at 37°C and 5% CO_2_ for 3 days.

For both conditions, cell viability was measured with CellTiter-Glo Luminescent Cell Viability Assay according to the manufacturer’s instruction. The percent cytotoxicity was calculated relative to the cell control wells (untreated cells) and normalized to the medium control wells (medium only), and the CC_50_ values were reported.

### Lethal influenza challenge mouse model

Female BALB/c mice (6–8 weeks old, Envigo) were randomized by body weight (*n* = 5 or 6 per dose group) and infected by intranasal (i.n.) administration of influenza virus A/Puerto Rico/8/1934 (H1N1; ATCC VR-95) under isoflurane anesthesia. Virus stock was diluted in PBS to inoculate 1,000 PFU (2 × LD_100_) in a total volume of 50 µL per mouse. VNT-101 was dissolved into vehicle (20% Captisol, San Diego, CA) by alternating vortexing and sonication, and then diluted to the appropriate concentrations to achieve doses of 30 mg/kg or 100 mg/kg in 100 µL volumes for administration by oral gavage (PO). VNT-101 was formulated in advance and stored at 4°C until the time of administration. Oseltamivir was included as a reference treatment group at 50 mg/kg (five times the approximate mouse equivalent of the human efficacious dose); oseltamivir was dissolved in PBS by alternating vortexing and sonication, and then diluted to the appropriate concentration to achieve doses of 50 mg/kg in 100 µL volumes for PO administration. Treatment was initiated either (i) immediately after infection (i.e., prophylaxis), (ii) at 48 hpi, or (iii) at 72 hpi and continued twice a day (BID) for a total of 5 days.

Primary readouts for the study were overall survival and percentage body weight loss, with mice monitored twice a day for 10 days post-infection for adverse clinical signs. Animals with a predefined clinical score of ≤2 or ≥25% reduction in body weight were considered moribund and were euthanized, in accordance with Novartis’ ethical guidelines. Percent body weight loss was calculated relative to day 0 and was plotted as a function of time, with the last observation carried forward (i.e., when an animal was removed from study due to body weight loss, the last body weight observation was carried forward for the remaining days on study). Percent survival was presented using a Kaplan-Meier survival plot. Graphs and statistical analyses were generated using GraphPad Prism 8.0.

### Quantitation of viral load in lung tissue homogenates

For the combination therapy study, to monitor both body weight loss/survival to the end of the study (day 10) and lung viral load reduction at the end of the treatment phase (day 8), each dose level had duplicate, parallel groups of animals. Each group for monitoring body weight and survival (odd-numbered groups) consisted of six animals. Each group for lung viral load analysis (even-numbered groups) consisted of five animals. The 10 groups had the following treatments: Groups 1 and 2, vehicle control; Groups 3 and 4, 30 mg/kg VNT-101 BID; Groups 5 and 6, 100 mg/kg VNT-101 BID; Groups 7 and 8, 50 mg/kg oseltamivir BID; and Groups 9 and 10, 30 mg/kg VNT-101 + 50 mg/kg oseltamivir BID.

Lung tissues were collected from animals in the even-numbered treatment groups (Groups 4, 6, 8, and 10; *n* = 5 per group) either at the end of the treatment phase (day 8) or at the time of death or euthanasia for moribund animals (if prior to day 8). Animals in vehicle control groups (Groups 1 and 2) died or were euthanized between day 3 and day 7. For this analysis, their data were combined into a single mean value. Lung viral loads were determined by plaque assay. Briefly, lung tissues were weighed and homogenized in 600 µL PBS, and 10-fold serial dilutions ranging from 10^−1^ to 10^−6^ were prepared in infection media (DMEM with 1 × penicillin-streptomycin solution) supplemented with 1 µg/mL TPCK-treated trypsin. Serial dilutions of lung homogenate (300 µL) were then used to infect MDCK cells plated the day before at 3 × 10^5^ cells/well in a 12-well plate (duplicate wells per dilution). After 1 h of incubation at 37°C, the dilutions of lung homogenate were removed, and cells were overlaid with 1 mL of 0.8% agarose in plaque assay medium (2 × MEM [without phenol red] with 1 × penicillin-streptomycin solution, 20 mM HEPES buffer, and 1 µg/mL TPCK-treated trypsin), allowed to solidify at room temperature, and incubated at 37°C with plates inverted. After 72 h, cells were fixed with 4% paraformaldehyde (PFA) for 1 h at room temperature. PFA was then aspirated, the agar was removed, and 1 mL of 0.1% crystal violet in 10% ethanol was added to each well and incubated for 30 min at room temperature. Excess crystal violet staining solution was washed away with water, and plates were allowed to air dry at room temperature. Finally, plaques were counted and used to calculate PFU/g of lung tissue for each animal.

## Data Availability

The data that support the findings of this study are available from the corresponding author upon reasonable request. The co-structure of VNT-101 complexed with recombinant NP from influenza A/Puerto Rico/8/1934 is deposited in the RCSB Protein Data Bank (PDB Accession #9OUG).

## SUPPLEMENTAL MATERIAL

Supplemental material aac.01149-25-s0001.docx Supplemental methods; Fig. S1 to S4; Tables S1 and S2.

ASM does not own the copyrights to Supplemental Material that may be linked to, or accessed through, an article. The authors have granted ASM a non-exclusive, world-wide license to publish the Supplemental Material files. Please contact the corresponding author directly for reuse.
