# Supplementary material for: Identification and characterization of a novel inhibitor of influenza A virus that acts by blocking nucleoprotein oligomerization
Source: Antimicrob Agents Chemother. 2025 Dec 19;70(2):e01149-25. doi: 10.1128/aac.01149-25 (PMC12888856; doi:10.1128/aac.01149-25)
Supplement: Supplemental material — Supplemental methods; Fig. S1 to S4; Tables S1 and S2. [file aac.01149-25-s0001.docx]

# Supplemental Information

**Title: Identification and characterization of a novel inhibitor of Influenza A virus that acts by blocking nucleoprotein oligomerization**

**Authors:** Vincent H. J. Leonard, Dianna B. Vidales, Benjamin R. Taft, Matthew J. Hesse, Patrick S. Lee, Mulugeta Mamo, Dirksen E. Bussiere, Karen C. Wolff, Kelli L. Kuhen, Laura Wedel, Ellena Growcott, Colin Osborne, Cassio P. Octaviani, Pinghan Huang, Chien-Te Kent Tseng, Johanna R. Abend, Kelly A. Wong, Weidong Zhong, David C. Tully, Don Ganem*

# **Supplemental Methods**

# **Gel filtration analysis of NP oligomerization**

**Methods:** Recombinant influenza virus A/Puerto Rico/8/1934 (H1N1) NP (amino acids 8-498, 6-histadine (His)-tagged, 56.5 kDa, 20.8 mg/ml stock) was expressed in *E. coli* and purified over HisTrap (Cytiva) and heparin columns. Superdex 200 Increase size exclusion chromatography (SEC) column (Cytiva) was equilibrated with 3 ml SEC buffer (20 mM sodium phosphate, pH 7, 500 mM sodium chloride [to favor trimerization of NP], 1 mM tris[2-carboxyethyl]phosphine [TCEP]) at a flow rate of 0.05 ml/min. Reaction samples were prepared with 25 µM NP in SEC buffer and treated with VNT-725 compound at 0.01, 1, and 100 µM in a total volume of 45 µl for 1 hour at rotating room temperature. Samples were then loaded into mass spectrometry tubes and run on the pre-equilibrated SEC column with molecular weight markers. Data was analyzed using Unicorn software.

# **Lethal influenza challenge mouse model – prophylaxis study**

**Methods:** Female BALB/c mice (6-8 weeks old) were randomized by body weight (n=6 per dose group) and infected by intranasal administration of 1000 PFU (2×LD_100_) influenza virus A/Puerto Rico/8/1934 (H1N1). VNT-101 was dissolved into vehicle (20% Captisol, San Diego, CA) and diluted to the appropriate concentrations to achieve doses of 1, 3, 10, or 30 mg/kg in 100 µl volumes for administration by oral gavage (PO); only data from the 30 mg/kg VNT-101 dose group is reproduced here for the convenience of the reader. Oseltamivir was included as a reference treatment group at 50 mg/kg (5 times the approximate mouse equivalent of the human efficacious dose). Treatment was initiated immediately after infection (ie, prophylaxis) and continued twice a day (BID) for a total of 5 days.

Primary readouts for the study were overall survival and percentage body weight loss (with the last body weight observation carried forward), with mice monitored twice a day for 10 days post-infection for adverse clinical signs (Figure S2). Animals with a predefined clinical score of ≤ 2, or ≥ 25% reduction in body weight were considered moribund and were euthanized, in accordance with Novartis’ ethical guidelines. On Day 3 post-infection, plasma samples were collected via retro-orbital (RO) bleeds from half of the mice (n=3) in each of the VNT-101 dose groups at 0.5 hr post-dose (left RO) and 12 hr post-dose (right RO), and from the remaining mice (n=3) in each VNT-101 dose group at 6 hr post-dose (left RO). Samples were analyzed by LC-MS/MS. A summary of the PK parameters is shown in Table S1.

# **In vitro resistance selection studies**

**Additional methods for multi-segment RT-PCR amplification, deep sequencing, and analysis:** Total RNA was extracted from influenza virus-containing culture supernatants using the QIAamp Viral RNA Mini Kit (Qiagen, cat #52906) according to the manufacturer’s instructions. The eight segments of the influenza virus genome were reverse transcribed and PCR amplified in a single reaction using Superscript III One-Step RT-PCR System with Platinum Taq High Fidelity DNA Polymerase (Life Technologies, cat #12574-035) according to the manufacturer’s instructions and previously published methods [1]. Amplicons were purified using Agencourt AMPure XP beads (Beckman Coulter, cat #A63881) in 96-well plate format according to manufacturer’s instructions with the exception that bead, wash, and elution volumes were optimized empirically. The concentration of purified DNA was determined by spectrophotometer (Nanodrop or Qubit) and samples were analyzed by agarose gel with comparison to the unpurified amplicon. AMPure XP beads exclude fragments below 500 base pairs (bp) to reduce background signal caused by defective interfering virus particles. Viral cDNA amplicons were prepared for next generation sequencing using Nextera DNA sample preparation kits (Illumina, cat #FC-121-1030) according to the manufacturer’s instructions. Following library preparation, samples were purified and concentration determined as above. In order to optimize the amplicon size, the standard Pippin Prep protocol (Sage Science, cat #CDF1510) was run and size selection was completed for 650 bp +/- 15%. The final sample was purified using DNA Clean & Concentrator-5 (Zymo Research, cat #D4014) Clean and Concentrate according to the manufacturer’s instructions. MiSeq reactions were prepared as described in Preparing DNA Libraries for Sequencing (Illumina).

For each sample, raw Illumina FASTQ sequence files were aligned to the influenza A/Puerto Rico/8/1934 (GenBank assembly GCF_000865725.1) reference nucleotide sequence using Bowtie 2, v.2.1.6 [2] and an in silico size selection was performed to exclude data from fragments smaller than 500 bp (parameters: --very-sensitive-local -N 1 -I 0 -X 1000). The resulting SAM files were parsed using in-house custom software written in Python (version 2.7.11). Base calls with quality scores less than 66 (phred+33 encoding) were removed. Frequencies of observed codons were calculated at each equivalent position in the proteome. For each position, codons that represented non-synonymous amino acid changes relative to the passage-equivalent DMSO samples, and that were present with at least 5% frequency among aligned reads with a minimum of 100 reads supporting the change, were reported.

# **Fig S1**


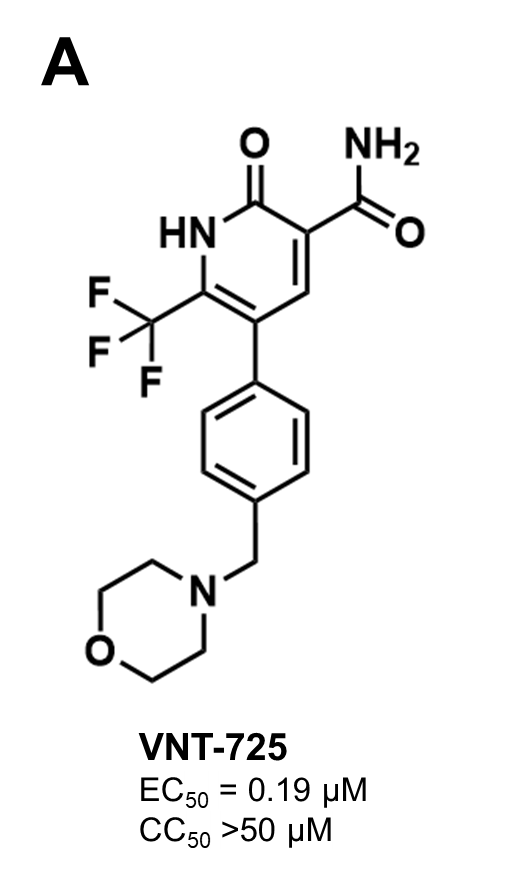


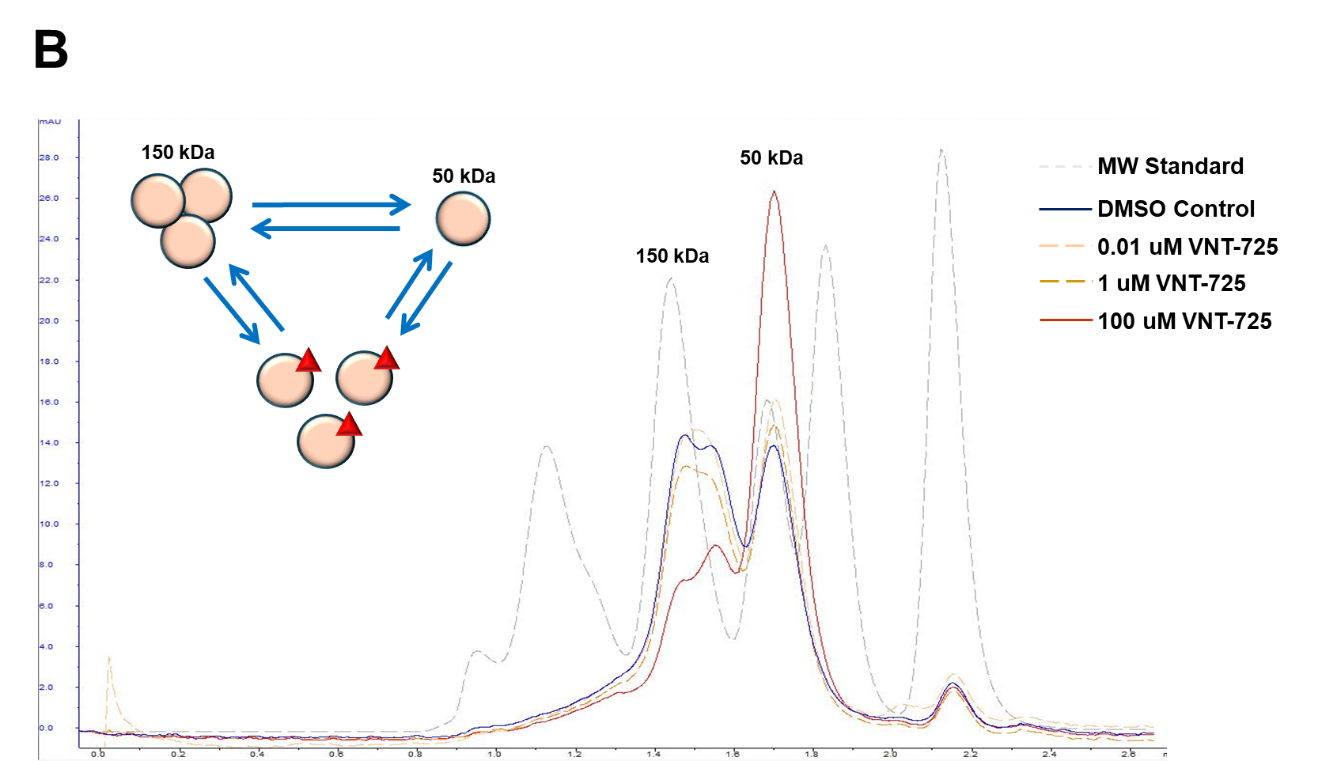


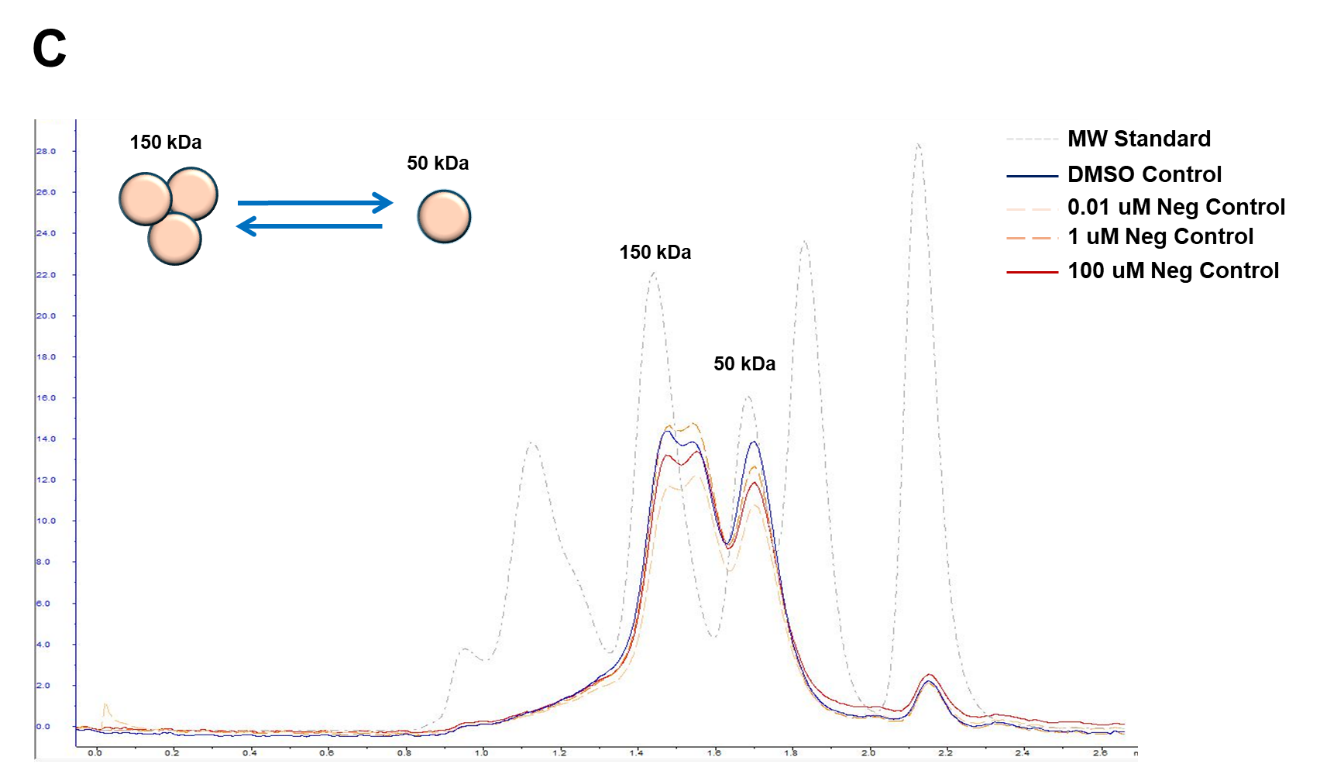


Figure S1. VNT-725 disrupts NP oligomerization by gel filtration analysis. (**A**) Structure and activity summary of VNT-725. (**B**) Shift of NP trimers (150 kDa) to monomers (50 kDa) in the presence of increasing concentrations of VNT-725, demonstrating disruption of NP oligomerization. (**C**) Specificity control for Panel B showing equilibrium of NP trimers (150 kDa) and monomers (50 kDa) in the presence of increasing concentrations of a chemically unrelated negative control compound that targets influenza A virus endonuclease (PA) protein, not NP. No shift from trimer to monomer peak is observed with escalating concentrations of the control compound.

Abbreviations: NP, nucleoprotein; MW, molecular weight; DMSO, dimethyl sulfoxide; kDa, kilodaltons; Neg, negative

# **Fig S2**


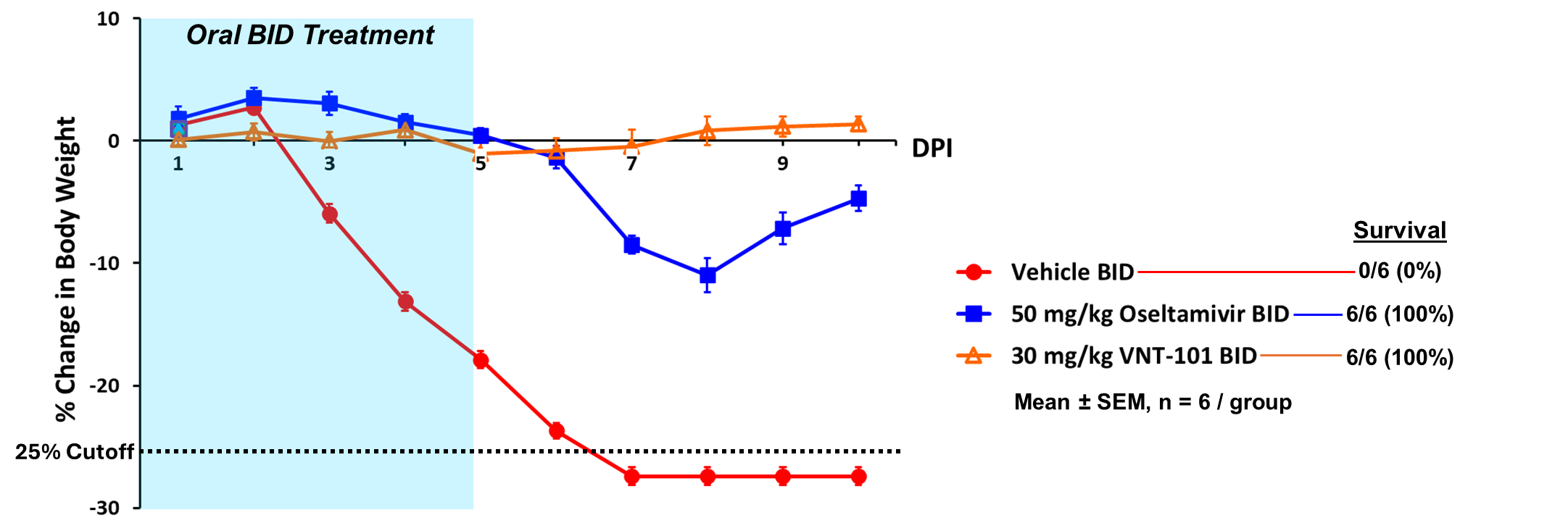


Figure S2. Change in body weight and percent survival with VNT-101 prophylaxis. Percent change in body weight is calculated based on initial measurement taken on Day 0 (with the last body weight observation carried forward). X-axis indicates days post-infection up to end of study (Day 10). Blue highlighted area indicates duration of treatment with VNT-101 or oseltamivir (up to Day 5 post-infection). Dotted line denotes 25% body weight loss cutoff, at which point animals were euthanized in accordance with ethical guidelines.

Abbreviations: DPI, days post infection; BID, twice a day; SEM, standard error of the mean.

# **Fig S3**


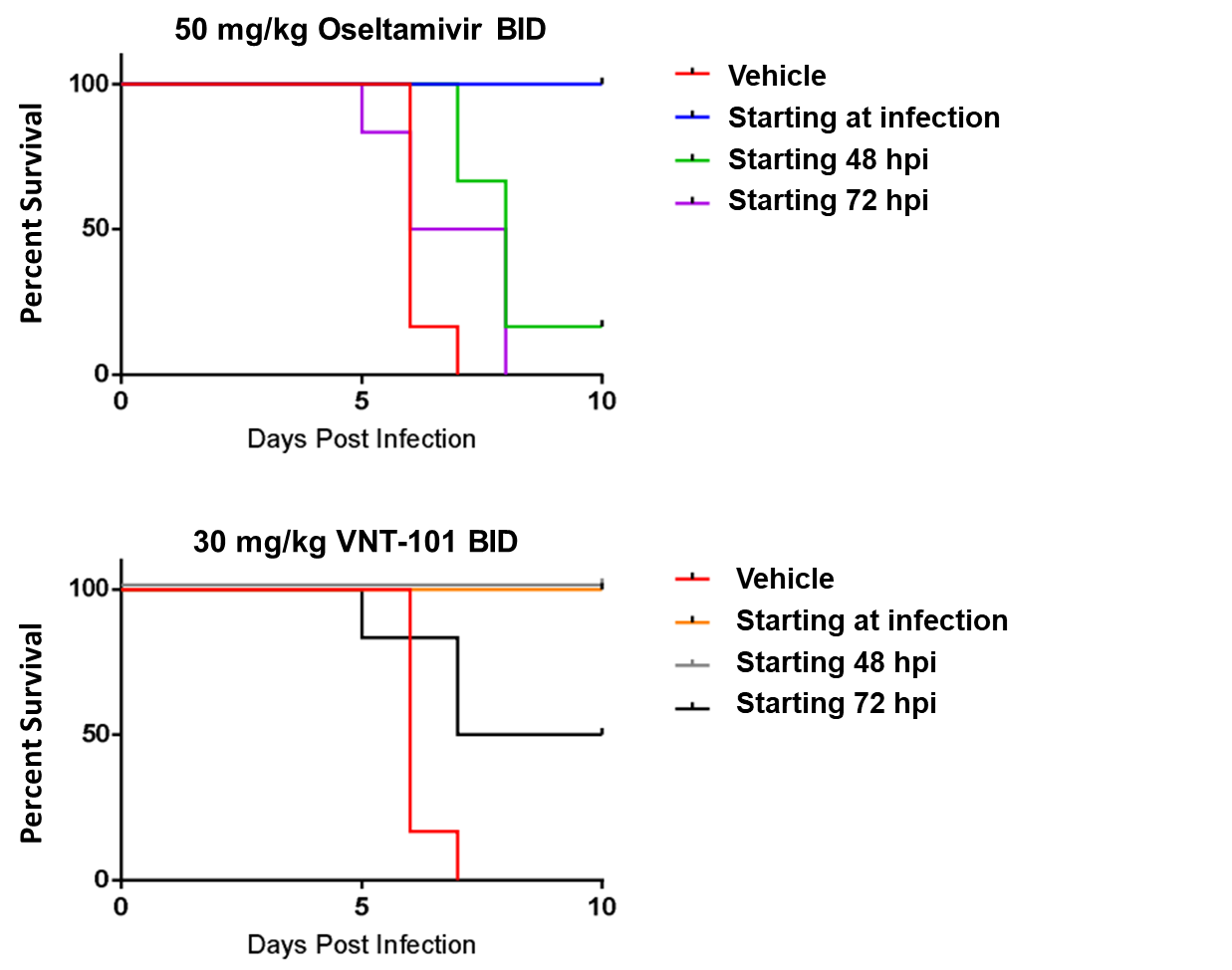


Figure S3. Percent survival with VNT-101 delayed treatment. Kaplan-Meier survival plot to show percent survival in each dose group (n=6). Survival curves for 30 mg/kg VNT-101 BID starting at infection and 48 hpi are identical (orange and grey lines overlap).

Abbreviations: BID, twice a day; hpi, hours post-infection.

# **Fig S4**


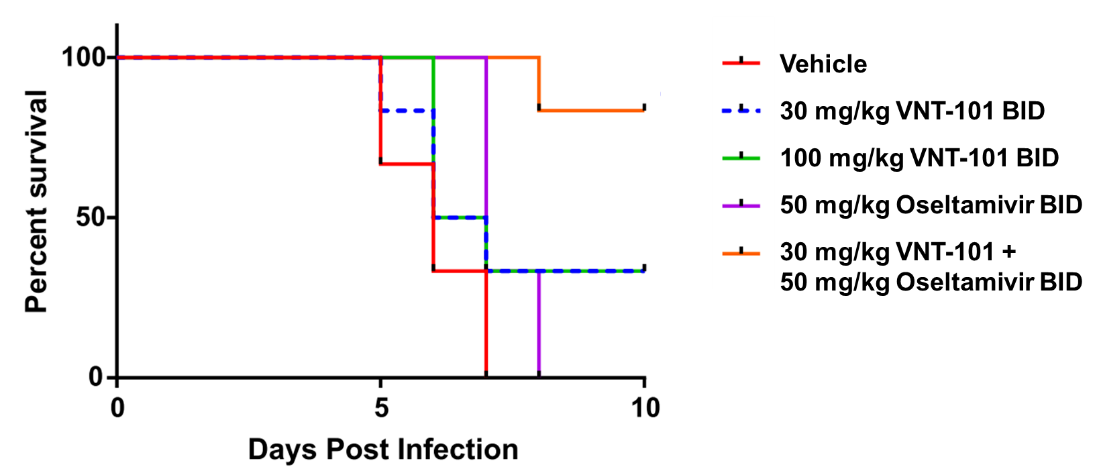


Figure S4. Percent survival with combination of VNT-101 plus oseltamivir. Kaplan-Meier survival plot to show percent survival in Groups 1, 3, 5, 7, and 9 (n=6 per group).

Abbreviations: BID, twice a day.

# **Table S1**

| VNT-101 BID Dose  (mg/kg) | C_max_  (μM) | C_min_  (μM) | fC_min_  (μM) | AUC  (μM *hr) | fAUC  (μM *hr) | AUC/  Dose | fC_min_/  EC_50_ | fAUC/  EC_50_ |
| --- | --- | --- | --- | --- | --- | --- | --- | --- |
| 30 | 31.3  ±5.32 | 3.49  ±1.35 | 0.10 | 105  ±30.2 | 3.045 | 3.5 | 20.24 | 609.0 |

Table S1. Summary of PK parameters for 30 mg/kg BID dose of VNT-101 in Balb/c mice at steady state. Data are presented as mean ± SD for n=3 animals. Plasma free AUC and free C_min_ were calculated based on percent plasma protein binding previously determined for VNT-101 (97.1%). VNT-101 activity against A/Puerto Rico/8/1934 (in parallel with murine model development and validation): mean EC_50_ value of 5 nM.

Abbreviations: AUC, area under plasma concentration-time curve; fAUC, free (unbound) AUC; fC_min_, free (unbound) C_min_; BID, twice a day; C_max_, maximum drug plasma concentration; C_min_, minimum drug plasma concentration; SD, standard deviation.

# **Table S2**

|  | **Co-structure of VNT-101 complexed with influenza NP** |
| --- | --- |
| **Data collection** |  |
| Space group | P41212 |
| Cell dimensions |  |
| *a*, *b*, *c* (Å) | 60.151, 60.151, 584.400 |
| α, β, γ (°) | 90.00, 90.00, 90.00 |
| Resolution (Å) | 47.48 – 2.33 (2.38 – 2.33) |
| *R*_merge_ | 0.2509 (2.241) |
| *CC1/2* | 0.996 (0.140) |
| *I /σI* | 4.98 (0.78) |
| Wilson B-factor | 41.03 |
| Completeness (%) | 99.60 (99.96) |
| Total observations | 583215 (27530) |
| Unique observations | 97505 (5322) |
| Redundancy | 6.0 (5.2) |
| **Refinement** |  |
| Resolution (Å) | 46.44 – 2.33 (2.38 – 2.33) |
| Number of reflections | 97505 (5322) |
| R_work_ / R_free_ | 0.2666 / 0.3160 (0.3507 / 0.3778) |
| No. of atoms |  |
| Protein | 10,808 |
| Ligand/additive | 64 |
| Water | 151 |
| Mean B-factors (Å^2^) |  |
| Protein | 54.53 |
| Ligand/additive | 67.60 |
| Water | 44.74 |
| R.m.s. deviations |  |
| Bond lengths (Å) | 0.008 |
| Bond angles (°) | 1.110 |

Table S2. X-ray crystallographic data collection and refinement statistics. Each dataset was collected from a single crystal. Values in parentheses are for highest-resolution shell.

Abbreviations: No., number; NP, nucleoprotein.

# **References**

| [1] | B. Zhou and D. E. Wentworth, “Influenza A virus molecular virology techniques,” *Methods Mol Biol,* pp. 175-92, 2012. |
| --- | --- |
| [2] | B. Langmead and S. L. Salzberg, “Fast gapped-read alignment with Bowtie 2,” *Nat Methods,* pp. 357-9, 2012. |
